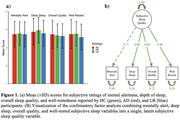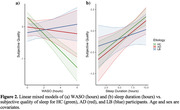# Misperception of night‐to‐night sleep quality in Alzheimer’s and Lewy body disease

**DOI:** 10.1002/alz.090809

**Published:** 2025-01-09

**Authors:** Alex Washburn, Joseph R. Winer, America Romero, Maya Yutsis, Victor W. Henderson, Kathleen L. Poston, Jamie M Zeitzer, Elizabeth Mormino

**Affiliations:** ^1^ Bowdoin College, Brunswick, ME USA; ^2^ Stanford University School of Medicine, Stanford, CA USA; ^3^ Veterans Affairs Palo Alto Health Care System, Palo Alto, CA USA; ^4^ Stanford University, Stanford, CA USA

## Abstract

**Background:**

Objective sleep measures obtained from actigraphy (wrist‐worn accelerometry) reveal sleep disruption patterns and may serve as indicators of neurodegenerative disease. However, whether individuals’ subjective account of their sleep quality corresponds to objective sleep measurements from the previous night is an area little studied. In particular, whether neurodegenerative disease modifies these associations is unknown.

**Method:**

N=111 participants including healthy controls (HC) and individuals with Alzheimer’s disease (AD) and Lewy body (LB) disease were recruited from the Stanford Alzheimer’s Disease Center and the Stanford Aging and Memory Study, with a mean of 6.3 ± 1.4 days of paired actigraphy/questionnaire data. Each morning, participants rated their overall sleep quality, sleep depth, well‐restedness, and mental alertness on a 0‐4 Likert scale, which were combined into a latent subjective sleep score using confirmatory factor analysis (Figure 1). Nightly sleep duration and wake after sleep onset (WASO, time awake after falling asleep) were objective actigraphy‐derived variables of interest. Linear mixed models were used to assess differences in the relationship between objective sleep variables and the subjective sleep score between disease etiologies.

**Result:**

Across all participants, subjective sleep scores were associated with the previous night’s objective recording. However, this association showed an interaction with etiology such that increasing WASO predicted reduced subjective sleep quality in HC participants, whereas for participants with AD and LB, subjective sleep quality did not relate to WASO (Figure 2a). In contrast, subjective sleep quality was predicted similarly by sleep duration in AD, LB, and HC participants: as sleep duration increased, subjective sleep quality increased (Figure 2b). These relationships also held when binning by cognitive impairment status (normal, mild cognitive impairment, dementia) or MoCA score rather than etiology.

**Conclusion:**

A significant difference in how objective sleep disruption predicts subjective sleep quality for AD and LB participants relative to HC participants suggests that individuals' perception of their night‐to‐night sleep quality may be impaired in the context of neurodegenerative disease.